# Infectious diseases and immunity: special reference to major histocompatibility complex.

**DOI:** 10.3201/eid0301.970105

**Published:** 1997

**Authors:** N Singh, S Agrawal, A K Rastogi

**Affiliations:** Department of Biochemistry, Central Drug Research Institute, Lucknow, India. root@cscdri.ren.nic.in

## Abstract

Human leukocyte antigens (HLAs) are an inherent system of alloantigens, which are the products of genes of the major histocompatibility complex (MHC). These genes span a region of approximately 4 centimorgans on the short arm of human chromosome 6 at band p 21.3 and encode the HLA class I and class II antigens, which play a central role in cell-to-cell interaction in the immune system. These antigens interact with the antigen-specific cell surface receptors of T lymphocytes (TCR) thus causing activation of the lymphocytes and the resulting immune response. Class I antigens restrict cytotoxic T-cell (CD8+) function thus killing viral infected targets, while class II antigens are involved in presentation of exogenous antigens to T-helper cells (CD4+) by antigen presenting cells (APC). The APC processes the antigens, and the immunogenic peptide is then presented at the cell surface along with the MHC molecule for recognition by the TCR. Since the MHC molecules play a central role in regulating the immune response, they may have an important role in controlling resistance and susceptibility to diseases. In this review we have highlighted studies conducted to look for an association between HLA and infectious diseases; such studies have had a variable degree of success because the pathogenesis of different diseases varies widely, and most diseases have a polygenic etiology.


### Infectious Diseases and Immunity: Special Reference to Major Histocompatibility Complex

**Neeloo Singh,* S. Agrawal,† and A.K. Rastogi***
*Department of Biochemistry, Central Drug Research Institute, Lucknow, India; †Department of Medical Genetics, Sanjay Gandhi Post Graduate Institute of Medical Sciences, Lucknow, India

Human leukocyte antigens (HLAs) are an inherent system of alloantigens, which are the products of genes of the major histocompatibility complex (MHC). These genes span a region of approximately 4 centimorgans on the short arm of human chromosome 6 at band p 21.3 and encode the HLA class I and class II antigens, which play a central role in cell-to-cell interaction in the immune system. These antigens interact with the antigen-specific cell surface receptors of T lymphocytes (TCR) thus causing activation of the lymphocytes and the resulting immune response. Class I antigens restrict cytotoxic T-cell (CD8+) function thus killing viral infected targets, while class II antigens are involved in presentation of exogenous antigens to T-helper cells (CD4+) by antigen presenting cells (APC). The APC processes the antigens, and the immunogenic peptide is then presented at the cell surface along with the MHC molecule for recognition by the TCR. Since the MHC molecules play a central role in regulating the immune response, they may have an important role in controlling resistance and susceptibility to diseases. In this review we have highlighted studies conducted to look for an association between HLA and infectious diseases; such studies have had a variable degree of success because the pathogenesis of different diseases varies widely, and most diseases have a polygenic etiology.

### Major Histocompatibility Complex (MHC) and Immune Response

Because of its remarkable power to deal with infection, the immune system is central to the prevention and control of infectious disease. Immune responsiveness is affected, even controlled, by gene products of the major histocompatibility system (1). Many diseases are associated with human leukocyte antigens (HLAs) (2,3). Moreover, in some infectious diseases (4-6), the host immune reactivity, which is responsible for the pathologic manifestation of disease, has been correlated with HLA specificities.

The discovery of the human MHC dates from the mid 1950s when leukoagglutinating antibodies were found in the sera of patients who received multiple transfusions and in the sera of 20% to 30% of multiparous women. In humans, the entire histocompatibility complex is termed the HLA complex. Genes coding for HLAs occupy a segment of approximately 4 centimorgans on the short arm of chromosome 6. The HLA-A, -B, and -C genetic loci determine class I antigens; HLA-DR, -DP, -DQ genetic loci determine class II antigens. Class I antigens are found on virtually every human cell; class II antigens are found chiefly on the surfaces of immunocompetent cells, including macrophages/monocytes, resting T lymphocytes, activated T lymphocytes, and particularly B lymphocytes.

The MHC molecule provides a context for the recognition of antigens by T lymphocytes. The polymorphic binding site of MHC class I and class II molecules is composed of a ß-pleated sheet flanked by two alpha helixes. They form a groove that accommodates one single microbial peptide ligand.

MHC class I molecules bind to peptides produced by the intracellular degradation of viral proteins and display them on the cell surface for recognition by CD8+ T lymphocytes. A class of white blood cells, the CD8 T lymphocytes, bear receptors specific for the HLA class I antigens

Address for Correspondence: Neeloo Singh, Department of Biochemistry, Central Drug Research Institute, Lucknow, India; fax: 91-522-223-405; e-mail: root@cscdri.ren.nic.in.

and route pathogens such as viruses. Surface expression of class I MHC molecules depends on the availability of peptides that bind MHC molecules in the endoplasmic reticulum. A peptide transporter, associated with antigen processing (TAP), plays an important role in maintaining adequate levels of peptide (7). The transporter is a heterodimer encoded by two genes, TAP1 and TAP2, located in the MHC class II region. TAP genes belong to the adenosine triphosphate (ATP) binding cassette super family of transport proteins, which have two ATP-binding cassette domains and two transmembrane domains. TAP genes are polymorphic (8), and allelic MHC differences may be associated with disease by altering the peptides that bind class I MHC molecules. Since human TAP genes are located between HLA-DP and HLA-DQ, TAP alleles could result in an apparent disease association with class II HLA alleles. Class I, i.e., HLA-A, -B, and -C molecules, play an important role in viral infections in the lysis of target cells by cytotoxic killer T lymphocytes.

MHC class II molecules are highly polymorphic membrane glycoproteins that bind peptide fragments of proteins and display them for recognition by CD4+ T lymphocytes. The white blood cells known as CD4 T lymphocytes are of central importance in defeating the bacteria and other parasites that live within cells. The CD4 T lymphocytes are called helper T cells because they secrete substances that amplify and control virtually all aspects of immunity. These T cells have receptor molecules that can recognize one particular peptide-HLA class II antigen combination. The binding capability of any given peptide to MHC class II molecules depends on the primary sequence of the peptide and allelic variation of the amino acid residues in the binding site of the MHC receptor. Anchor residues defining allele-specific peptide motifs have been identified in the class II binding peptides. The proposed anchor residues combining with MHC pockets through their side chains seem to be a primary requirement for peptide-MHC interaction. The invariant chain (Ii) plays a critical role in the assembly, intracellular transport, and function of MHC class II molecules (9). In intracellular parasites (e.g., *Leishmania* infections of macrophages), it is the class II MHC molecules that specifically bind to receptors on these microbes.

### HLA Association with Infectious Diseases

Infectious diseases are associated with impaired immunity. Some persons mount very effective immune responses when given vaccines, while others respond to vaccines poorly or not at all. The level of response is determined by several factors: intensity of infection, factors related to the intensity of the host immune response, T-cell state, T-cell function, and perhaps most important, the genetic factor that interacts with the other factors to determine the outcome of the disease. Infectious disease research is now focusing on genetic markers such as allelic forms of HLA molecules.

### HLA Association with Mycobacterial Infections

Genetic factors may control host responses to *Mycobacterium tuberculosis* (10-12). Several investigators have conducted population studies to determine an association between pulmonary tuberculosis (TB) and HLA specificities. HLA-DR2 is associated with the development of multibacillary forms of both TB and leprosy (13,14); molecular subtyping of DR2 showed that the majority of the allele in patients and controls was DRB1*1501 and DRB1*1502. The frequency of these molecular subtypes of DR2 in patients was not skewed, suggesting that the entire DR2 molecule or its closely linked gene(s) may govern patient susceptibility to pulmonary TB and, particularly, to drug-resistant TB. When the three-dimensional structure of the HLA-DR molecule is elucidated (15), sequencing of class II alleles in patients with pulmonary TB and drug-resistant TB could identify an amino acid residue(s) critical for the binding of a *M. tuberculosis*-derived pathogenic peptide(s) responsible for the detrimental or protective immune response.

HLA alleles also modulate the immune response that determines the form of leprosy (a heterogeneous disease caused by *Mycobacterium leprae)* that develops in each patient (16,17). At one pole of the spectrum of leprosy are the multibacillary lepromatous leprosy (LL) patients, who are anergic to the antigens of *M. leprae,* and at the other extreme are the paucibacillary tuberculoid leprosy (TT) patients, who exhibit a good cell-mediated immune response. Humoral immunity is present throughout the spectrum but does not seem to provide protection. Between the two poles are patients with intermediate features as seen in the borderline lepromatous, borderline leprosy (BB), and borderline tuberculoid forms (18). An increased frequency of HLA-DR2 and -DQ1 in LL patients (19) and of HLA-DR3 in TT patients has been reported (20). These antigens can be further subdivided into alleles defined by their amino acid sequence. A single amino acid substitution may give rise to alleles with different immunologic properties. The allele DRB1*1501 showed a stronger association with LL patients than with TT patients ($p < 0.00001$). In addition, DQB1*0601 was found significantly more often in LL patients than in controls ($p < 0.00001$); DQA1*0103 was more frequent in the LL group than in the tuberculoid leprosy group; and DQA1*0102 was selectively increased in patients with borderline lepromatous leprosy (Table 1). However, DRB1*0701, DQB1*0201, and DQA1*0201 were decreased in LL patients compared with TT patients and controls, and DQB1*0503 was selectively decreased in TT patients, suggesting that these alleles might modulate the immune response that determines the form of leprosy that develops in each patient (21).

Table 1. Frequency of HLA class II alleles with significant differences between leprosy patients and healthy controls

| HLA alleles | Healthy controls N=47 N | % | Leprosy patients N=93 N | % | RR[a] | p |
|---|---|---|---|---|---|---|
| DRB1*15 | 10 | 21.3 | 70 | 75.3 | 11.3 | <0.00001 |
| DRB1*1501 | 6 | 12.8 | 49 | 52.7 | 7.6 | <0.00001 |
| DRB1*1502 | 5 | 10.6 | 26 | 27.9 | 3.2 | <0.05 |
| DRB5*0101 | 6 | 12.8 | 49 | 52.7 | 7.6 | <0.00001 |
| DRB5*0102 | 5 | 10.6 | 26 | 27.9 | 3.2 | <0.05 |
| DQA1*0102 | 9 | 19.1 | 38 | 40.9 | 2.9 | <0.05 |
| DQA1*0103 | 13 | 27.6 | 48 | 51.6 | 2.8 | <0.05 |
| DQB1*0601 | 8 | 17.0 | 56 | 60.2 | 7.4 | <0.00001 |
| DRB1*0404 | 5 | 10.6 | 0 | 0.0 | 0.04 | <0.01 |
| DRB1*0701 | 13 | 29.8 | 11 | 11.8 | 0.3 | <0.05 |
| DRB1*1401 | 4 | 8.5 | 0 | 0.0 | 0.005 | <0.05 |
| DQB1*0503 | 16 | 36.2 | 14 | 16.1 | 0.3 | <0.05 |

[a]RR = relative risk

### HLA Association with Parasitic Infections

Because there are significant differences between malaria-exposed and -unexposed populations in the frequencies of HLA genes at the A and B loci, the HLA complex may protect populations in endemic-disease areas who are exposed to malaria parasites. The adaptative mechanisms may be expressed by HLA-associated genes that control immune responsiveness to malaria antigens. The association between the HLA class I antigen HLA-B53 and protection from severe malaria has been well established (5). This link might be mediated by HLA class I restricted cytotoxic T lymphocytes (CTL) during the liver stage of the parasite's life cycle (22). The protective association between HLA-B53 and severe malaria was investigated by sequencing peptides eluted from this molecule before testing candidate epitopes from preerythrocytic-stage antigens of *Plasmodium falciparum* in biochemical and cellular assays. Among malaria-immune Africans, HLA-B53 restricted CTL recognized a conserved nonamerpeptide from liver stage-specific antigen, but no HLA-B53 restricted epitopes were identified in antigens from other stages (5). These findings indicate a possible molecular basis for this HLA disease association and support the candidacy of liver stage-specific antigen as a malaria vaccine component.

The association between HLA-DR/-DQ phenotypes and immune response to circumsporozoite protein of the human malaria parasite were investigated in Thai adults (23). Evidence suggests that human T- and B-cell responses to a major *P. falciparum* antigen (Pf RESA) in persons primed by repeated infections are genetically regulated (24). To associate T-cell and antibody responses with the donors' MHC class II genotypes, genomic HLA class II typing of DQ antigens of leukocytes from 145 donors living in endemic-disease regions of Africa were performed by restriction fragment length polymorphism (24). These data imply that the impact of MHC class II gene products on specific immune responses to Pf 155/ RESA epitopes is weak and hard to demonstrate in outbred human populations naturally primed by infection. The relationship between class II HLA and immune recognition of three candidate antigens for a vaccine against *P. falciparum* was investigated in persons extremely heterozygous for HLA class II alleles living in an endemic-disease area of West Africa (25). One class II DQA-DQB combination (serologic specificity DQw2) was particularly common among these persons. This haplotype was significantly associated with higher than average levels of antibody to a peptide epitope (EENV)6 of Pf RESA. There was little evidence of association between HLA class II genotype and cellular proliferation responses to the antigen tested.

The frequency of HLAs was studied in 62 patients with scabies and 27 patients with cutaneous leishmaniasis to evaluate the role of HLA antigens as genetic markers in the pathogenesis of these parasitic skin diseases. A significant statistical association was found between HLA-A11 antigen and scabies and between HLA-A11, -B5, and -B7 antigens and diffuse cutaneous leishmaniasis (26). In another study, 24 families with one or more cases of localized cutaneous leishmaniasis from an endemic-disease region with the highest incidence of localized cutaneous leishmaniasis in Venezuela were typed for HLA-A, -B, -C, -DR, and -DQ antigens and complement factors. The parental HLA haplotypes segregated at random among healthy and affected siblings, but in backcross families significantly higher frequencies of HLA-A28, -Bw22 or -DQw8 were present in infected compared with healthy siblings (27). In addition, HLA-B15 showed a higher frequency among healthy siblings. Haplotypes Bw22, DR11, DQw7 were also significantly more frequent in infected than in healthy siblings. No HLA linkage with a putative localized cutaneous leishmaniasis susceptibility gene(s) could be demonstrated in this study (27). A case/control comparison of 26 unrelated localized cutaneous leishmaniasis patients and healthy persons of the same ethnic origin confirmed the association of HLA-Bw22 and -DQw3 with this disease. The relative risk reached 12.5 for Bw22 and 4.55 for DQw3. HLA-DQw3 apparently makes the major contribution as a genetic risk factor for localized cutaneous leishmaniasis at the population level. In another study, a statistically significant association was found between HLA-B5 and -DR3 and schistosomiasis (28).

A study of the association of HLA class I antigen frequencies in 52 patients with kala-azar and 222 unrelated healthy controls in Iran found HLA-A26 to be statistically significant ($p = 0.004$) (29). This indicates a high risk of contracting the disease for HLA-A26 positive persons and a remarkable influence of this antigen on the prevalence rate of kala-azar.

The significance of susceptibility/protection correlations between HLA and parasitic diseases has been established by serologic typing methods. To improve the accuracy of MHC-disease associations, we have used a DNA-based HLA typing method, namely polymerase chain reaction with sequence specific oligonucleotide probes, for the molecular typing of kala-azar patients in India (30). To study the possible association at the molecular level of HLA class I (A and B) as well as class II (DR) antigens in kala-azar patients, we typed patients with kala-azar by polymerase chain reaction with sequence specific oligonucleotide probes and compared the antigen frequencies with healthy family-based controls. On the basis of the distribution of alleles in each sample, percentage phenotype and genotype frequencies were calculated for both control and kala-azar patients. Statistical analysis using the Transmission Disequilibrium Test was carried out to assess the association of different HLA allelic specificities with kala-azar patients. No significant association between any of the HLA class I or class II antigens was found. We will conduct a linkage analysis study based on the data from typing the above-mentioned case/controls. The findings might lead to a new dimension in the study of HLA association with parasitic infections: genetic markers, such as HLA, that are sufficiently polymorphic (as measured by their heterozygosities) can be used in linkage and association analysis to detect Mendelian segregation underlying disease phenotypes (31).

Comprehensive analysis of HLA associations with infectious diseases has allowed precise definition of susceptibility and protective alleles in large populations of different ethnic origins. Of great interest in the fine dissection of molecular mechanisms leading to parasitic diseases, these studies also provide the genetic basis for identification of the subset of persons at risk for subsequent infection. Infectious diseases may have exerted significant pressure on the development and maintenance of HLA polymorphism (32). Widespread and frequently fatal parasitic diseases such as malaria have selectively maintained certain gene frequencies in endemic-disease areas (33).

Although HLA associations with parasitic diseases have provided clues to pathogenesis, the molecular basis of these associations has not yet been defined. The determinant selection hypothesis, which states that associations result from the ability of a particular HLA type to present a critical antigenic peptide, has been difficult to investigate because, for most disease associations, the relevant antigen is unknown. Recently, the identification of characteristic sequence features in peptides eluted from HLA class I molecules (34,35) suggested that the relevant antigen might be identifiable by assessing cellular immune responses to peptides containing such motifs among antigens that are candidates for mediating

HLA disease associations. With the development of modern techniques of the HLA assembly assay (36), relevant peptides can be synthesized; it can then be determined whether they have any function as CTL epitopes during immune responses. Such studies will elucidate the HLA associations with parasitic infections and the molecular basis of these associations and facilitate the development of vaccines for these infectious diseases.

### HLA Association with Viral Infections

The associations of viral diseases with HLA alleles have not been studied extensively. However, mechanisms by which HLA molecules determine the immune response to viral peptides have been well studied as part of efforts to develop safe and efficient virus vaccines. Successful development of vaccines against viral infections depends on the ability of inactivated and live virus vaccines to induce a humoral immune response and produce antiviral neutralization antibodies. Additionally, virus vaccines that induce a cellular immune response leading to the destruction of virus-infected cells by CD8+ CTLs may be needed to provide protection against some viral infections. Antiviral CD8+ CTLs are induced by viral peptides presented within the peptide binding grooves of HLA class I molecules on the surface of infected cells. Studies in the last decade have provided an insight into the presentation of viral peptides by HLA class I molecules to CD8+ T cells.

*Herpesvirus saimiri*, an oncogenic, lymphotropic, gamma-herpesvirus, transforms human and simian T cells in vitro and causes lymphomas and leukemias in various species of New World primates. An open reading frame of the *H. saimiri* genome encodes a heavily glycosylated protein that is secreted and binds to heterodimeric MHC class II HLA-DR molecules (37). These results indicate that the open reading frame can function as an immunomodulator that may contribute to the immunopathology of *H. saimiri* infection.

Cytotoxic T cells that recognize dengue virus peptides have been reported (38). Analysis of HLA class I haplotype-restricted peptides showed that HLA-A2 and -A68 motifs were abundant compared with nonpeptides with HLA-A24, -B8, and -B53 motifs. Studies by Zeng et al. (39) suggest that the T-cell response to dengue virus is restricted by the HLA-DR15 allele. Becker (40) developed an approach to priming antiviral CD8+ CTLs that may provide cellular immune protection from flavivirus infection without inducing the humoral immune response associated with dengue fever shock syndrome. He proposed using synthetic flavivirus peptides with an amino acid motif to fit with the HLA class I peptide binding group of HLA haplotypes prevalent in a given population in an endemic-disease area as an immunogen. These synthetic viral peptides may be introduced into the human skin by using a lotion containing the peptides (Peplotion) and substances capable of enhancing the penetration of these peptides into the skin to reach Langerhans cells. The peptide-treated Langerhans cells, professional antigen presenting cells, may bind the synthetic viral peptides by their HLA class I peptide binding grooves. Antigens carrying Langerhans cells can migrate and induce the cellular immune response in the lymph nodes.

Transmission of human immunodeficiency virus 1 (HIV-1) from an infected woman to her offspring during gestation and delivery is influenced by the infant's MHC class II DRB1 alleles. Forty-six HIV-infected infants and 63 seroreverting infants, born with passively acquired anti-HIV antibodies but not becoming detectably infected, were typed by an automated nucleotide-sequence-based technique (41). One or more DR-13 alleles, including DRB1*1301, 1302, and 1303 were found in 15.2% of those becoming HIV-infected and 31.7% of seroreverting infants ($p = 0.048$); this association was influenced by ethnicity. Upon examining for other allelic associations, only the DR2 allele DRB1*1501 was associated with seroreversion in Caucasian infants. Among these infants, the DRB1*03011 allele was positively associated with HIV infection.

Molecular mimicry, where structural properties borne by a pathogen "imitate" or "simulate" molecules of the host, also appears to be an important mechanism in the association of HLA molecules with viral disease. Molecular mimicry takes different forms in the molecular biology of HIV-1 (42). Molecular mimicry between HIV envelope proteins and HLA class II molecules may lead to autoimmunity against CD4+ T cell expressing class II molecules (43). Bisset (44) states that both the HIV-1 gp 120 envelope and *Mycoplasma genitalium* adhesion proteins share an area of significant similarity with the CD4-binding site of the class II MHC proteins. Interaction with this triad could contribute to T-

cell dysfunction, T-cell depletion, Th1-cell–Th2-cell shift, B-cell proliferation, hyperglobulinemia, and antigen-presenting cell dysfunction.

HLA-DR has been evaluated as a marker for immune response related to human cytomegalovirus infection (45); this virus plays a role in chronic inflammatory reaction in inflammatory abdominal aortic aneurysm. In the fibrously thickened adventitia of this aneurysm, human cytomegalovirus-infected cells and HLA-DR positive cells were more frequently encountered than in that of atherosclerotic aneurysms and control cases ($p < 0.01$).

An estimated 250 million people throughout the world are chronically infected with hepatitis B virus, the primary cause of chronic hepatitis, cirrhosis, and hepatocellular carcinoma in endemic-disease areas (46,47). Because HLA class I antigens contain viral peptides, they may be important targets for immune mediated hepatocytolysis by CD8+ CTLs in hepatitis B virus infection (48). Davenport et al. (49) have shown that HLA-DR13 is associated with resistance to hepatitis B virus infection. Prognosis may be quite different among patients infected with hepatitis C virus: a chronic liver disease occurs in half the patients, while the other half exhibits no signs of histologic progression of liver damage. The host immune responses may play an important role in such different outcomes. To identify human CTL epitopes in the NS3 region of hepatitis C virus, Kurokohchi et al. (50) modified an approach using recombinant protein and the ability of short peptides to bind to class I MHC molecules. They identified a cytotoxic T-cell epitope presented by HLA-A2 in the hepatitis C virus NS3 region. A study conducted by Peano et al. (51) establishes that HLA-DR5 antigen appears as a protective factor against a severe outcome of hepatitis C virus infection.

Epstein-Barr virus, a member of the herpesvirus family, has been associated with virus replication (infectious mononucleosis, oral hairy leukoplakia) as well as neoplastic conditions such as nasopharyngeal carcinoma, B-cell lymphoma, and Hodgkin disease associated with viral latency. An influence of CTL response on Epstein-Barr virus evolution was first suggested by the finding that virus isolates from highly HLA-A11–positive Asian populations were specifically mutated in two immunodominant A11 restricted CTL epitopes (52). Additionally, B35.01-restricted CTL responses in white donors reproducibly map to a single peptide epitope (53). However, most Epstein-Barr virus isolates from a population where B35.01 was prevalent (in the Gambia) either retained the CTL epitope sequence or carried a mutation that conserved antigenicity; changes leading to reduced antigenicity were found in only a minority of cases. Two epitopes for Epstein-Barr virus specific CTLs restricted by the common allele HLA-B7 were identified by Hill et al. (54).

The level of serum HLA class I antigens markedly increases during the course of viral infections such as those caused by cytomegalovirus, hepatitis B virus, hepatitis C virus, HIV-1, and varicella-zoster virus (55-57). During HIV-1 infection, the level of serum HLA class I antigens correlates with disease stage and represents a good prognostic marker of disease progression (55).

### HLA Association with Bacterial Infections

Vaccines based on recombinant attenuated bacteria represent a potentially safe and effective immunization strategy. A carrier system was developed by Verjans et al. (58) to analyze in vitro whether foreign T-cell epitopes, inserted in the outer membrane protein PhoE of *Escherichia coli* and expressed by recombinant bacteria, are efficiently processed and presented through HLA class I and II molecules by infected human macrophages.

A well-defined HLA-B27 restricted cytotoxic T-cell epitope and an HLA-DR53 restricted T-helper epitope of the fusion protein of measles virus were genetically inserted in a surface-exposed region of PhoE, and the chimeric proteins were expressed in *E. coli* and *Salmonella typhimurium*. Macrophages infected with recombinant bacteria presented the T-helper epitope to a specific CD4+ T-cell clone but failed to present the CTL epitope to the specific CD8+ T-cell clone. Phagocytic processing of intact bacteria within infected macrophages was essential for antigen presentation by HLA class II. Nascent HLA class II molecules were also required for the presentation of the T-helper epitope to the CD4+ T-cell clone by infected macrophages.

HLA associations may also link various diseases; for example the HLA-B27 association for ankylosing spondylitis, Reiter disease, reactive arthropathy, and acute anterior uveitis indicate that these disorders may share a pathogenic pathway. According to the molecular mimicry hypothesis, antigens carried by a particular pathogen may resemble a certain HLA allomorph. As the person carrying this allomorph is unresponsive to it, it is susceptible to the disease caused by the pathogen. For example, some investigators believe that one of the antigens of *Klebsiella* resembles HLA-B27 and that pathogen is responsible for ankylosing spondylitis (59). In most patients who have an acute attack of anterior uveitis, a common ocular disease characterized by inflammation of the iris and ciliary body, the only clues to the pathogenesis of this disease are its close association with the genetic marker HLA-B27 and the likely triggering role of a variety of gram-negative bacteria (60). HLA-B27 acute anterior uveitis appears to be a distinct clinical entity frequently associated with the seronegative arthropathies, such as ankylosing spondylitis and Reiter syndrome.

Sasazuki (61) showed that low responsiveness to streptococcal cell wall antigen was inherited as an HLA-linked dominant trait. The immune suppression gene for streptococcal cell wall was in strong linkage disequilibrium with particular alleles of the HLA-DQ locus. This shows that the HLA-linked immune suppression genes exist in humans to control low response to natural antigens.

Table 2 lists the associations that have been established between various HLA factors and certain infectious diseases. Only the antigens showing statistically significant associations are indicated. Because some persons are unresponsive to certain critical epitopes of the pathogens presumably responsible for certain infectious diseases, particular HLA alleles occur more frequently in patients with certain infectious diseases than in healthy persons; therefore, researchers associate these diseases with certain HLA alleles. This article has summarized the findings from population genetic analysis and from studies of the association of immune response mechanisms of infectious diseases and HLA.

Table 2. Association between human leukocyte antigen (HLA) and some infectious diseases

| Disease | HLA Association |
|---|---|
| *Bacterial* | |
| Ankylosing spondylitis | B27 |
| Reiter disease | B27 |
| Acute anterior uveitis | B7 |
| *Mycobacterial* | |
| Tuberculosis and leprosy | DR2 |
| (multibacillary forms) | (DRB1*1501, 1502) |
| lepromatous leprosy | DR2 and DQ1 |
| paucibacillary tuberculoid | DR3 |
| *Viral* | |
| Dengue fever virus | DR15 |
| Human immunodeficiency | DR13 |
| virus 1 | (DRB1*1301, 1302, 1303) |
| | DR2 |
| | (DRB1*1501) |
| | DRB1*03011 |
| Hepatitis B virus | DR13 |
| Hepatitis C virus | A2 |
| | DR5 |
| Epstein-Barr virus | B35.01 |
| | A11 |
| | B7 |
| *Parasitic* | |
| Malaria | B53 |
| Scabies | A11 |
| Diffuse cutaneous leishmaniasis | A11, B5, B7 |
| Localized cutaneous | A28, Bw22, DQw8 |
| leishmaniasis | Bw22, DR11, Qw7 |
| | Bw22, Dqw3 |
| Schistosomiasis | B5, DR3 |
| Visceral leishmaniasis | A26 |

### Acknowledgments

We are grateful to Dr. Derek Middleton for critically reading the manuscript and Dr. Martin Curran and Mr. J.C. Sharma for their help in the literature survey. This work was supported by a grant from the Department of Science and Technology, India (No. SR/OY/MB-10/92).

Dr. Singh is a scientist in the Department of Biochemistry, Central Drug Research Institute, Lucknow. Her research interest is visceral leishmaniasis, or kala-azar, as it is known in eastern India, where the disease is now epidemic. She is developing PCR-based diagnostics for the disease focusing on kinetoplast DNA, studying the molecular mechanisms of drug resistance, and striving to answer the most important question: are host genetic factors, like HLA, involved in susceptibility to kala-azar in India.

### References

1. McDevitt HO, Bodmer WF. Histocompatibility antigens, immune responsiveness and susceptibility to disease. Am J Med 1972;52:1-8.
2. Dausset J, Svejgaard A, editors. HLA and disease. Copenhagen:Munksgaard, 1977.

3. Stastny P, Ball EJ, Dry PJ, Nunez G. The human immune response region (HLA-D) and disease susceptibility. Immunol Rev 1983;70:111-54.
4. Tiwari JL, Terasaki PI, editors. HLA and disease association. New York: Springer 1985.
5. Hill AVS, Elvin J, Wills AC, Aidoo M, Allsopp CEM, Gotch FM, et al. Molecular analysis of the asso-ciation of HLA-B53 and resistance to severe malaria. Nature 1992;360:434-9.
6. Assaad-Khalil SH, Helmy MAS, Zaki A, Mikhail MM, El-Hai MA, El-Sawy M. Some genetic, clinical and immunologic interrelations in schistosomiasis mansoni. Ann Biol Clin 1993;51:619-26.
7. Colonna M, Bresnahan M, Bahram S, Strominger JL, Spies T. Allelic variants of the human putative peptide transporter involved in antigen processing. Proc Natl Acad Sci USA 1992;89:3932-6 .
8. Powis SH, Tonks S, Mockridge I, Kelly AP, Bodmer JG, Trowsdale J. Alleles and haplotypes of the MHC-encoded ABC transporters TAP1 and TAP2. Immunogenetics 1993;37:266-73.
9. Sant AJ, Miller J. MHC class II antigen processing: biology of invariant chain. Curr Opin Immunol 1994;6:57-63.
10. Kallmann FJ, Reisner D. Twin studies on the significance of genetic factors in tuberculosis. American Review of Tuberculosis 1943;47:549-74.
11. Bossik LJ. On the roles of hereditary and environment in the physiology and pathology of childhood tuberculosis. Proceedings of Maxim Gorky Medical Biology Research Institute (Moscow) 1934;19:111.
12. Nakamura RM, Tokunaga T. Strain difference of delayed type hypersensitivity to BCG and its genetic control in mice. Infect Immun 1978;78:657-64.
13. Rajalingam R, Mehra NK, Jain RC, Myneedu VP, Pande JN. Polymerase chain reaction-based sequence-specific oligonucleotide hybridization analysis of HLA class II antigens in pulmonary tuberculosis: relevance to chemotherapy and disease severity. J Infect Dis 1996;173:669-76.
14. Xu XP, Li SB, Wang CY, Li OH. Study on the association of HLA with pulmonary tuberculosis. Immunol Invest 1986;15:327-32.
15. Brown JH, Jardetzky TS, Gorga JC, Stern LJ, Urban RG, Strominger JL, et al. Three dimensional structure of the human class II histocompatibility antigen HLA-DR1. Nature 1993;364:33-9.
16. van Eden W, de Vries R, D'Amaro J. HLA-DR associated genetic control of the type of leprosy in a population from Surinam. Hum Immunol 1982;4:343-50.
17. Serjeantson S. HLA and susceptibility to leprosy. Immunol Rev 1983;70:89-112.
18. Ridley D, Jopling W. Classification of leprosy according to immunity. A five groups system. International Journal of Leprosy 1996;34:255-732.
19. van Eden W, Gonzales N, de Vries R, Convit J, van Rood J. HLA-linked control of predisposition of lepromatous leprosy. Infectious Diseases 1985;151:9-14 .
20. Gorodezky C, Flores J, Arevalo N. Tuberculoid leprosy in Mexicans is associated with HLA-DR3. Lepr Rev 1987;58:401-6.
21. Rani R, Fernandez-Vina MA, Zaheer SA, Beena KR, Stastny P. Study of HLA class II alleles by PCR oligotyping in leprosy patients from North India. Tissue Antigens 1993;42:133-7.
22. Hill AVS, Allsopp CEM, Kwiatkowski D, Anstey NM, Twumasi P, Rowe PA, et al. Common West African HLA antigens are associated with protection from severe malaria. Nature 1991;352:595-600 .
23. Brown AE, Chandanayingyong D, Fuggle SV, Webster HK. Immune responses to the circumsporozoite protein of Plasmodium falciparum in relation to HLA-DR type. Tissue Antigens 1989;34:200-4 .
24. Troye-Blomberg, Olerup O, Larsson A, Sjoberg K, Perlmann N, Riley E, et al. Failure to detect MHC class II associations of the human immune response induced by repeated malaria infections to the *Plasmodium falciparum* antigen Pf 155/RESA. Int Immunol 1991;3:1043-51.
25. Riley EM, Olerup O, Troye-Blomberg M. The immune recognition of malaria antigens. Parasitology Today 1991;7:5-11.
26. Morsy TA, Romia SA, al-Ganayni GA, Abu-Zakham AA, al-Shazly AM, Rezk RA. Histocompatibility antigens (HLA) in Egyptians with two parasitic skin diseases (scabies and leishmaniasis). J Egypt Soc Parasitol 1990;20:565-72.
27. Lara ML, Layrisse Z, Scorza JV, García E, Stoikaw Z, Granados J, et al. Immunogenetics of human American cutaneous leishmaniasis-study of HLA haplotypes in 24 families from Venezuela. Human Immunol 1991;30:129-35.
28. Assaad-Khalil SH, Helmy MAS, Zaki A, Mikhail MM, El-Hai MA, El-Sawy M. Some genetic, clinical and immunologic interrelations in schistosomiasis mansoni. Ann Biol Clin 1993;51:619-26.
29. Faghiri Z, Tabei SZ, Taheri F. Study of the association of HLA class I antigens with kala azar. Hum Hered 1995;45:258-61.
30. Singh N, Sundar S, Williams F, Curran MD, Rastogi A, Agrawal S, et al. Molecular typing of HLA class II DR antigens in Indian kala azar patients. Trop Med Int Health. In press.
31. Elston RC. Linkage and association to genetic markers Exp Clinical Immunogenetics 1995;12:3, 129-40.
32. Potts WK, Wakeland EK. The maintenance of MHC polymorphism. Immunology Today 1990;11:39-42.
33. Osaba D, Dick HM, Voller A, Goosen TJ, Goosen T, Draper C, et al. Role of the HLA complex in the antibody response to malaria under natural conditions. Immunogenetics 1979;8:323-38.
34. Falik K, Rotzsoke O, Stevanovic S, Jung G, Rammensee H. Allele-specific motifs revealed by sequencing of self peptides eluted from MHC molecules. Nature 1991;351:290-6.
35. Jardetsky TS, Lane WS, Robinson RA, Madden DR, Wiley DC. Identification of self peptides bound to purified HLA-B27. Nature 1991;353:326-9.
36. Elvin J, Cerundolo V, Elliot T, Townsend A. Quantitative assay of peptide dependent class I assembly. Eur J Immunol 1991;21:2025-31 .

37. Yao Z, Maraskovsky E, Spriggs MK, Cohen JI, Armitage RJ, Alderson MR. Herpesvirus saimiri open reading frame 14, a protein encoded by T lymphotropic herpes-virus, binds to MHC class II molecules and stimulates T cell proliferation. J Immunol 1996;156:3260-6.
38. Becker Y. Computer simulations to predict the availability of peptides with known HLA class I motifs generated by proteolysis of dengue fever virus (DFV) type I structural and nonstructural proteins in infected cells. Virus Genes 1995;10:195-203.
39. Zeng L, Kurane I, Okamoto Y, Ennis FA, Brinton MA. Identification of amino acids involved in recognition by dengue virus NS3-specific, HLA-DR15-restricted cytotoxic CD4+ T-cell clones. J Virol 1996;70:3108-17.
40. Becker Y. Dengue fever virus and Japanese encephalitis virus synthetic peptides, with motifs to fit HLA class I haplotypes prevalent in human populations in endemic regions, can be used for application to skin Langerhans cells to prime antiviral CD8+ cytotoxic T cells (CTLs)—a novel approach to the protection of humans. Virus Genes 1994;9:33-45.
41. Winchester R, Chen Y, Rose S, Selby J, Borkowsky W. Major histocompatibility complex class II DR alleles DRB1*1501 and those encoding HLA-DR13 are preferentially associated with a diminution in maternally transmitted human immunodeficiency virus 1 infection in different ethnic groups: determination by an automated sequence-based typing method. Proc Natl Acad Sci 1995;92:12374-8.
42. Bjork RL Jr. HIV-1: seven facets of functional molecular mimicry. Immunol Lett 1991;28:91-6.
43. Kamoun M, Zerva L, Sloan S, Zmijewski C, Monos D, Trinchieri G. Induction of HLA class II molecules on human T cells: relationship to immunoregulation and the pathogenesis of AIDS. DNA Cell Biol 1992;11:265-8.
44. Bisset LR. Molecular mimicry in the pathogenesis of AIDS: the HIV/MHC/mycoplasma triangle. Med Hypotheses 1994;43:388-96.
45. Yonemitsu Y, Nakagawa K, Tanaka S, Mori R, Sugimachi K, Sueishi K. In situ detection of frequent and active infection of human cytomegalovirus in inflammatory abdominal aortic aneurysms: possible pathogenic role in sustained chronic inflammatory reaction. Lab Invest 1996;74:723-36.
46. Beasley RP, Hwang LY, Lin CC, Chien CC. Hepatocellular carcinoma and hepatitis B virus. Lancet 1981;ii:1129-33.
47. Chen DS. From hepatitis to hepatoma: lessons learned from type B hepatitis. Science 1993;262:369-70.
48. Tsai SL, Chen MH, Yeh CT, Chu CM, Lin AN, Chiou FH, et al. Purification and characterization of a naturally processed hepatitis B virus peptide recognized by CD8+ cytotoxic T lymphocytes. J Clin Invest 1996;97:577-84.
49. Davenport MP, Quinn CL, Chicz RM, Green BN, Willis AC, Lane WS, et al. Naturally processed peptides from two disease resistance associated HLA -DR13 alleles show related sequence motifs and the effects of the dimorphism at position 86 of the HLA-DR beta chain. Proc Natl Acad Sci 1995;92:6567-71.
50. Kurokochi K, Akatsuka T, Pendleton CD, Takamizawa A, Nishioka M, Battegay M, et al. Use of recombinant protein to identify a motif-negative human cytotoxic T-cell epitope presented by HLA-A2 in the hepatitis C virus NS3 region. J Virol 1996;70:232-40 .
51. Peano G, Menardi G, Ponzetto A, Fenoglio LM. HLA-DR5 antigen. A genetic factor influencing the outcome of hepatitis C virus infection? Arch Intern Med 1994;154:23:2733-6.
52. Wolf H, Bogedain C, Schwarzmann F. Epstein-Barr virus and its interaction with the host. Intervirology 1993;35:26-39.
53. Lee SP, Morgan S, Skinner J, Thomas WA, Jones SR, Sutton J, et al. Epstein-Barr virus isolates with the major HLA B35.01-restricted cytotoxic T lymphocyte epitope are prevalent in a highly B35.01-positive African population. Eur J Immunol 1995;25:102-10.
54. Hill A, Worth A, Elliott T, Rowland-Jones S, Brooks J, Rickinson A, et al. Characterization of two Epstein-Barr virus epitopes restricted by HLA-B7. Eur J Immunol 1995;25:18-24.
55. Ferrone S, Yamamura M, Grosse-Wilde H, Pouletty P. Proceedings of the 11th International Histocompatibility Workshop. In: Tsuji K, Aizawa M, Sasazuki T, editors. Oxford Science Publications 1992;1057-61.
56. Alvarez-Cermeno JC, Casado C, Villar LM, Ferreira A, Varela JM, Dominguem, et al. Soluble class I antigens (SHLA) in CSF and serum of patients with HIV infection. Acta Neurol Scand 1990;82:14-6.
57. Puppo F, Scudeletti M, Indiveri F, Ferrone S. Serum HLA class I antigens: markers and modulators of an immune response? Immunology Today 1995; 16:124-7.
58. Verjans GM, Janssen R, UytdeHaag FG, van Doornik CE, Tommassen J. Intracellular processing and presentation of T cell epitopes, expressed by recombinant *Escherichia coli* and *Salmonella typhimurium*, to human T cells. Eur J Immun 1995; 25:405-10.
59. Scofield RH, Kurien B, Gross T, Warren WB, Harley JB. HLA-B27 binding of peptide from its own sequence and similar peptides from bacteria: implications for spondyloarthopathies. Lancet 1995; 345:1542-4.
60. Wakefield D, Montanaro A, McCluskey P. Acute anterior uveitis and HLA-B27. Surv Opthalmol 1991;36:223-32.
61. Sasazuki T. HLA-linked immune suppression genes. Jinrui Idengaku Zasshi 1990;35:1-13.
